# *In vivo* monitoring of chemically evoked activity patterns in the rat trigeminal ganglion

**DOI:** 10.3389/fnsys.2013.00064

**Published:** 2013-10-08

**Authors:** Matthias Lübbert, Jessica Kyereme, Markus Rothermel, Christian H. Wetzel, Klaus-Peter Hoffmann, Hanns Hatt

**Affiliations:** ^1^Department of Cell Physiology, Ruhr University BochumBochum, Germany; ^2^Brain Institute and Department of Neurobiology and Anatomy, University of UtahSalt Lake City, UT, USA; ^3^Molecular Neurosciences, Department of Psychiatry and Psychotherapy, University of RegensburgRegensburg, Germany; ^4^Department of Zoology and Neurobiology, Ruhr University BochumBochum, Germany

**Keywords:** trigeminal sensory neurons, trigeminal system, trigeminal chemoperception, odorant detection, chemesthesis, voltage-sensitive dye imaging

## Abstract

Albeit lacking a sense of smell, anosmic patients maintain a reduced ability to distinguish different volatile chemicals by relying exclusively on their trigeminal system (TS). To elucidate differences in the neuronal representation of these volatile substances in the TS, we performed voltage-sensitive dye imaging (VSDI) in the rat trigeminal ganglion (TG) *in vivo*. We demonstrated that stimulus-specific patterns of bioelectrical activity occur within the TG upon nasal administration of ten different volatile chemicals. With regard to spatial differences between the evoked trigeminal response patterns, these substances could be sorted into three groups. Signal intensity and onset latencies were also dependent on the administered stimulus and its concentration. We conclude that particular compounds detected by the TS are represented by (1) a specific spatial response pattern, (2) the signal intensity, and (3) onset latencies within the pattern. Jointly, these trigeminal representations may contribute to the surprisingly high discriminative skills of anosmic patients.

## Introduction

The trigeminal system (TS) provides facial mechanosensation (including discriminative touch), thermosensation, proprioception, chemoreception, and nociception (Lazarov, 2002; Viana, 2011). Stimuli activating the TS therefore include mechanical forces, temperatures, and certain chemicals. Thus, the TS fulfills important functions such as the detection and avoidance of potentially noxious stimuli within facial regions or sensing of the nasal air flow during breathing.

Due to its chemosensory abilities, the TS contributes to overall gustatory and olfactory sensations and most odorants stimulate cells belonging to the trigeminal and the olfactory system (OS) (Tucker, 1971; Doty, 1975; Doty et al., 1978; Silver and Moulton, 1982). Generally, chemical stimulation of trigeminal sensory afferents leads to different sensations (mostly stinging, burning, or cooling) that are triggered by activation of several polymodal receptors like transient receptors potential (TRP) channels that are activated by noxious stimuli e.g., harmful temperatures (Vay et al., 2012), various chemical ligands (Islam, 2011), and divalent cations (Ahern et al., 2005; Luebbert et al., 2010).

Interestingly, besides its function as a somatosensory and an alerting system, the TS provides the ability to discriminate between different chemicals. Although anosmic patients have lost fine odor discrimination skills, they are still able to roughly distinguish between different odor categories and even some stereoisomers (Laska et al., 1993; Thuerauf et al., 1999). Consistently, anosmic mice with a disrupted cyclic nucleotide-gated channel subunit 2 retained the ability to detect and discriminate several odorants and pheromones in a behavioral paradigm (Lin et al., 2004). Although several studies addressed the question how volatile chemicals are represented in higher brain regions (for review see Boyle et al., 2007; Hummel et al., 2009; Albrecht et al., 2010; Lundström et al., 2011), nearly nothing is known about the impact of the trigeminal ganglia, housing the somata of trigeminal sensory neurons, on the overall representation of different volatiles.

Topographically, somata of the mandibular branch are housed within the posterolateral portion of the TG, while cell bodies of the ophthalmic branch are localized anteromedially, and those of the maxillary branches are interposed in-between (Lazarov, 2002). The localization of TG neurons innervating the nasal cavity was further analyzed by viral tracing (Rothermel et al., 2011). Different kinds of TG neurons differ with regard to their morphological, histological, and histochemical properties but only few studies addressed questions regarding the chemosensory probabilities of TG neurons innervating distinct facial areas. It was nevertheless reported that TRPM8 is mainly expressed in neurons of the maxillary branch, whereas TRPV1 and TRPA1 seem to be homogenously expressed across all three trigeminal branches (Kobayashi et al., 2005). Furthermore, trigeminal projections innervating the nose were reported to feature a higher sensitivity for capsaicin and menthol than cutaneous projections (Damann et al., 2006). Although these findings underline the physiological impact of nasal trigeminal chemosensation, it is still unclear how the TS contributes to overall trigeminal sensations evoked by volatile chemicals and how different stimuli are represented in the TS. Only one study provided first insights in the representation of a small set of substances at the level of the trigeminal ganglion (TG) (Rothermel et al., 2011). The study focused on the establishment of a decerebration protocol, enabling direct monitoring of neuronal activity from large areas of the rat TG by using voltage-sensitive dye imaging (VSDI) *in vivo*. The authors verified that neuronal activity monitored within the TG closely correlates with single cell membrane potentials. In spite of the novel experimental approach, this study was based upon a limited set of only four substances which were always administered in the same concentration.

Therefore, the first aim of the present study was to characterize the occurrence of stimulus-specific activity patterns in the TG upon nasal administration of ten typical olfactory and trigeminal stimuli *in vivo*. The larger number of substances tested, gave rise to a substantially broader picture of the total variability of activity patterns occurring in the TG. The observed stimulus-induced activity patterns were analyzed by principle component analysis (PCA). In this way, the activity patterns could be clustered into three different groups of substances. In addition, our data reveal that the strength and the onset latencies of the monitored signals are highly dependent on the administered stimulus. Furthermore, the administration of different concentrations of selected substances indicated that not only the kind but also the concentration of a given substance is represented within the TG. Summarized, we found representations of chemical volatiles in the TG which are characterized by specific patterns of spatial activity, signal-intensities, and onset latencies. The existence of such representations may underlie the surprisingly high discriminative skills of anosmic patients mediated by the TS.

## Materials and methods

### Animals

VSDI data were acquired from 32 adult male Wistar rats (Charles River Laboratories, Germany). Water and food were offered *ad libidum*. *In vivo* experiments were approved by the German Animal Care and Use Committee (reference number: 9.93.2.10.32.07.022) and were performed in accordance with the Deutsches Tierschutzgesetz (§ 8 Abs. 1 Tierschutzgesetz) and the NIH guidelines. All experiments involving animals were carried out in accordance with the European Union Community Council guidelines.

### Animal preparation to access the trigeminal ganglia *in vivo*

The surgery was performed as previously described (Rothermel et al., 2011). In brief, animals were initially anesthetized by an injection with chloral hydrate (intra peritoneal, 25% solution in saline, 400 mg kg^−1^). Lidocaine (10 mg/ml) was applied to all incisions and pressure points. During experiments, animals were fixed in a stereotactic apparatus. They were tracheotomized and artificially ventilated (50–75 cycles/min, 5–6.5 ml tidal volume; UGO BASILE, Italy). Anesthesia was maintained using isoflurane (1%). An electrocardiogram, the exhaled CO_2_ level, and the rectal temperature were continuously monitored (core temperature was held at 37.5°C). A craniotomy was performed and the hemispheres were gently removed to access the TG at the base of the skull (Stüttgen et al., 2006). Since the decerebration causes unconsciousness, the concentration of isofluran was decreased to less than 1% prior to starting the measurements in order to exclude interference with nociceptive channels (Matta et al., 2008). Measurements were performed for up to 8 h after decerebration. Animals were euthanized with an overdose of anesthetic after the experiments.

### Loading of the TG

Recordings were performed using the voltage-sensitive dye RH-1838 (Optical Imaging Inc., USA) that binds to the external surface of cellular membranes without interfering with the cells physiological functions. It has an absorption maximum within the range of 550 and 570 nm and alters the intensity of emitted light (680–690 nm) as a function of changes in membrane potential. For loading of the TG, a micropipette, pulled from borosilicate glass capillaries (1.17 × 1.50 × 100 mm, Science Products, Germany) using a horizontal puller (Zeitz Instruments, Germany), was filled with saline solution containing the voltage-sensitive dye RH-1838. Afterwards, small amounts of dye were injected into the ganglion at 15–20 locations (total injected volume ~500 and 1000 nl) evenly distributed across the ganglion by manually applying slight pressure via a syringe directly connected to the micropipette.

### VSDI

In order to monitor optical signals arising from the TG, we used an Imager 3001 (Optical Imaging Inc., USA). All signals were collected at 100 Hz by a Dalstar 1M60 CCD camera (Dalsa, USA), coupled with a tandem lens macroscope (50 mm/1.2 toward camera and 50 mm/1.2 toward subject). The TG was illuminated with 630 ± 10 nm light and emitted light was high-pass filtered with a cutoff at 665 nm using a dichroid filter system. Data acquisition was triggered by coincident heart beat and respirator phase to minimize artifacts occurring from small movements induced by these events.

### Stimulus application during VSDI-recordings

For nasal administration of volatile stimuli, we used an OL022 olfactometer (Burghart Medizintechnik, Germany). This olfactometer containing four vapor lines (flow rate 0.4 l/min). One of them was used as a control line (saturator tube contained *aqua dest*.), one line contained pure CO_2_ (33% final concentration), which was used as a positive stimulus. Saturator tubes of the remaining channels were filled with varying substances. Vapor lines merge with a moistened background channel (flow rate 1 l/min) terminating in a single tube. This was gently adjusted to the nostril ipsilaterally to the imaged ganglion, in order to ensure that stimuli were exclusively administered to the animal's nose. A reverse tracheotomy was adapted as previously described for mice (Silver and Moulton, 1982; Wachowiak and Cohen, 2001), in order to ensure a constant nasal passage of the stimulating air stream thus minimizing mechanically and thermally induced trigeminal activations by alterations in the air stream. Animals were ventilated via the lower tracheotomy tube. Stimuli were applied in a randomized order for 4.8 s with inter-stimulus intervals of 20 s. For mechanical stimulation, we used forceps to slightly pinch the animal's nose (~0.5–1 s) once during recording from the TG.

### Data analysis of VSDI-data

First, each pixel was divisively normalized to its mean value during the pre-stimulus period (i.e., *F*/*F*_0_). Afterwards, activation of each condition was compared to the mean of the two blank conditions and was expressed as the relative change in fluorescence (Δ*F*/*F* = *F*/*F*_0_ − *F*_*B*_/*F*_0*B*_). Significance of signals across trials was expressed as *z*-score, where *z* = Δ*F*/*F*/√(SEM(*F*/*F*_0_)^2^ + SEM(*F*_*B*_/*F*_0*B*_)^2^) (SEM = standard error of the mean). Activity patterns of individual subjects were obtained by averaging the main response interval (2–4.8 s after stimulus onset). The stability of activity maps across all 29 animals which were stimulated with CO_2_ (the remaining three were mechanically stimulated) was verified by statistical comparison of CO_2_ evoked activity monitored from the animals number 1–15 with activity monitored from the animals number 16–29 by a Mann–Whitney-*U*-test. Onset latencies were defined as the time points at which signals exceeded a *z*-score ≥2. Unless otherwise stated all VSDI-data are presented as the median +75th percentile/−25th percentile. Normal-distribution was tested by using the Kolmogorov-Smirnov test (KS-test). Since not all data sets were normally distributed, Mann-Whitney-*U*-test was used for statistical comparison of averaged signal-intensities and onset-latencies. Statistical testing and the PCA were performed by using standard algorithms in Matlab (The MathWorks, USA). Countour plots were created with SigmaPlot (Systat software, USA), box plot diagrams with Origin (OriginLab Corporation, USA). Numbers of repeated experiments are indicated as *n* = X animals.

## Results

### Nasal application of volatile substances induces bioelectrical activity in the TG

In this study, we used VSDI to monitor bioelectrical activity at the level of the TG of adult male wistar rats upon nasal stimulation with the volatile chemicals ethanol (EtOH), menthol, CO_2_, eugenol, allylisothiocyanate (AITC), geraniol, vanillin, helional, sandalore, and 2-phenylethanol (PEE). Compounds were prepared as 1 mM in aqueous solution, except of AITC (150 μM), EtOH (99.99%), and CO_2_ (33%).

The substances EtOH (*n* = 5), menthol (*n* = 6), AITC (*n* = 5), geraniol (*n* = 7), and eugenol (*n* = 6) were chosen for this experiment since they induce trigeminal sensations by activation or modulation of ligand- or voltage-gated channels (Trevisani et al., 2002; Clapham, 2003; Behrendt et al., 2004). Sandalore (*n* = 4) and helional (*n* = 5) are frequently used in fragrances, although until today no study described their effects on the TS. The substances vanillin (*n* = 4) and PEE (*n* = 5) were previously classified as pure olfactory stimuli (pure odors) reported to lack any trigeminal component (Doty et al., 1978; Radli and Wysocki, 1998; Cometto-Muñiz et al., 2005; Daiber et al., 2012). Interestingly, nasal administration of vanillin was shown to induce bioelectrical activity at the level of the TG *in vivo* (Rothermel et al., 2011) and was tested in order to further substantiate this finding. PEE was therefore used as a negative control. CO_2_ (*n* = 29) was shown to activate exclusively the TS and was therefore administered in each experiment as a positive control (Doty et al., 1978).

**Figure 1:**
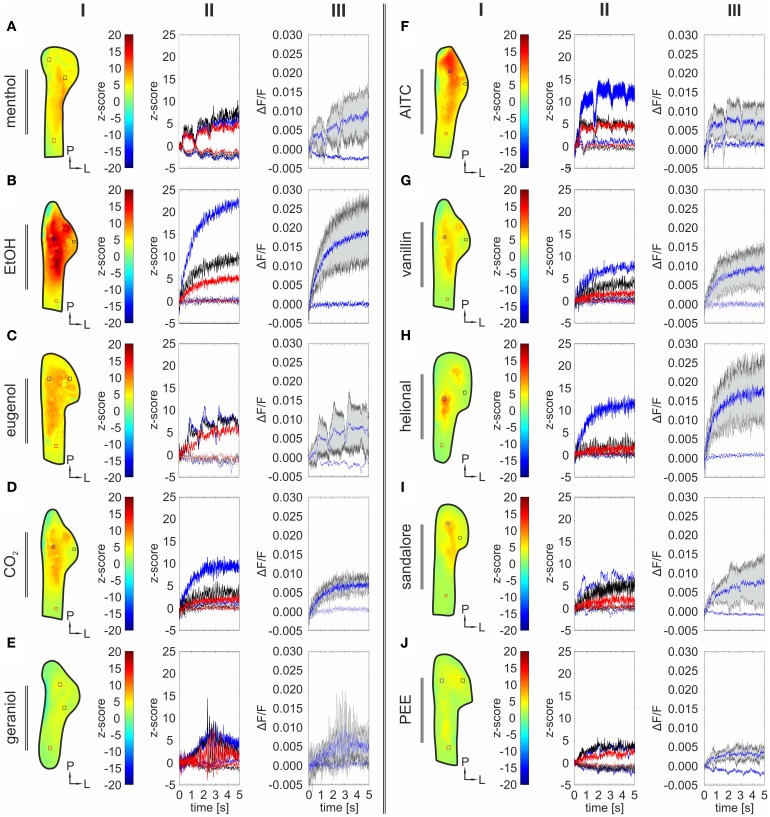
**Nasally administered compounds evoke bioelectrical activity in the rat TG *in vivo*.** The figure is arranged in columns indicated by roman numerals (I–III). Each row refers to one nasally administered stimulus: menthol **(A)**, EtOH **(B)**, eugenol **(C)**, CO_2_ **(D)**, geraniol **(E)**, AITC **(F)**, vanillin **(G)**, helional **(H)**, sandalore **(I)**, and PEE **(J)**. I: Representative *z*-score maps (2000–4800 ms average) depicting stimulus-induced activity patterns in the TG derived from experiments in single animals, respectively. ROI's referring to local time courses in II were manually selected, aiming to visualize the time course of *z*-score values within anterior (red square), lateral (black square), and postero-medial areas (blue-square). II: Local time course of *z*-score values monitored in the highlighted areas in **(I)**. Dotted lines display activity within the same ROI during blank administration (A.dest). Strongest signal-intensities were monitored within postero-medial areas (blue square). The intensities in more anterior (red square) and lateral (black square) areas were significantly smaller, although they still reached significant values (*z*-score ≥2). III: Local time course (Δ*F*/*F*) of activity monitored in postero-medial areas of the TG, marked in **(I)** by blue squares. Lightly gray highlighted areas represent variance across single trials (*n* = 3–5 trials). Dotted lines display activity within the same ROI during blank administration (A.dest).

In general, a high signal-to-noise ratio was attained in each experiment and signals were judged as statistically significant once exceeding a *z*-score ≥2. Signal onset latencies were defined as the interval between stimulus onset and detection of significant activity within a given range of interest (ROI). Interestingly, nasal application of all tested substances induced significant bioelectrical activity within the TG at the given concentrations. Exemplary *z*-score maps of bioelectrical activity including local-time courses of the monitored activity and *z*-score values in selected regions of interest are depicted in Figures 1A–J (II–III). In rare cases, we observed deflections in the local-time courses obtained from individual animals (Figures 1C,F) which seemed to be driven by the breathing or the respiration rhythm. These animals retained autonomous or spontaneous breathing, even after removal of the hemispheres. Breathing frequency in animals that revealed autonomous/spontaneous breathing occurred with an irregular frequency. The small movement correlating with single gasps may lead to these deflections that, in contrast to regular movements resulting from artificial respiration, could not be successfully filtered.

**Figure 2:**
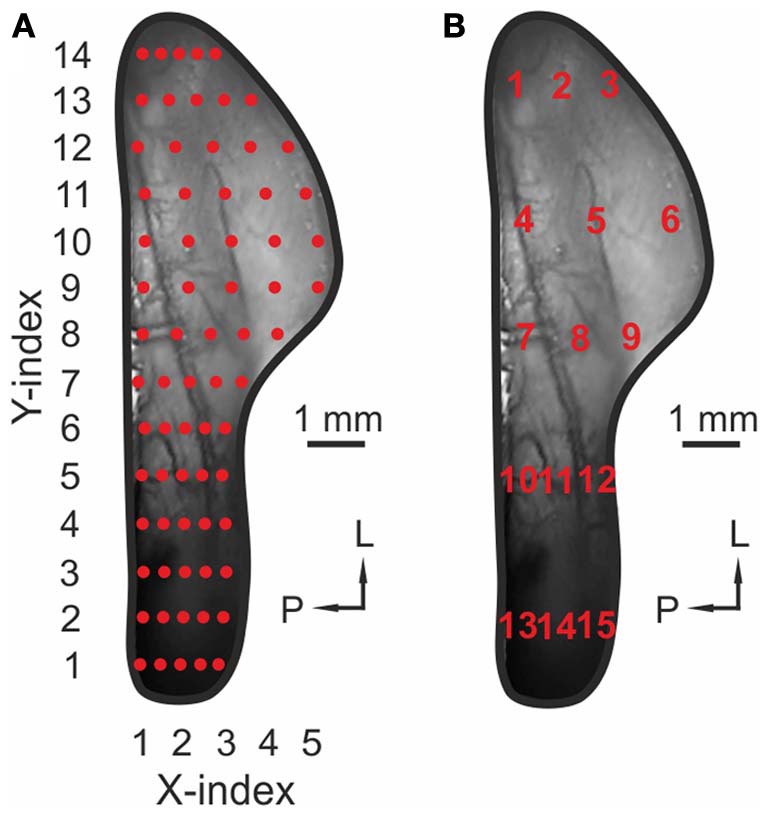
**Uniform distribution of ROIs across the TG. (A)** Image of a TG *in vivo*. Red markers, 70 ROIs used to create the contour plots in Figures 3–5. **(B)** Image of a TG *in vivo.* Red numbers (1–15): ROIs used to create bar diagrams in Figure 7.

**Figure 3:**
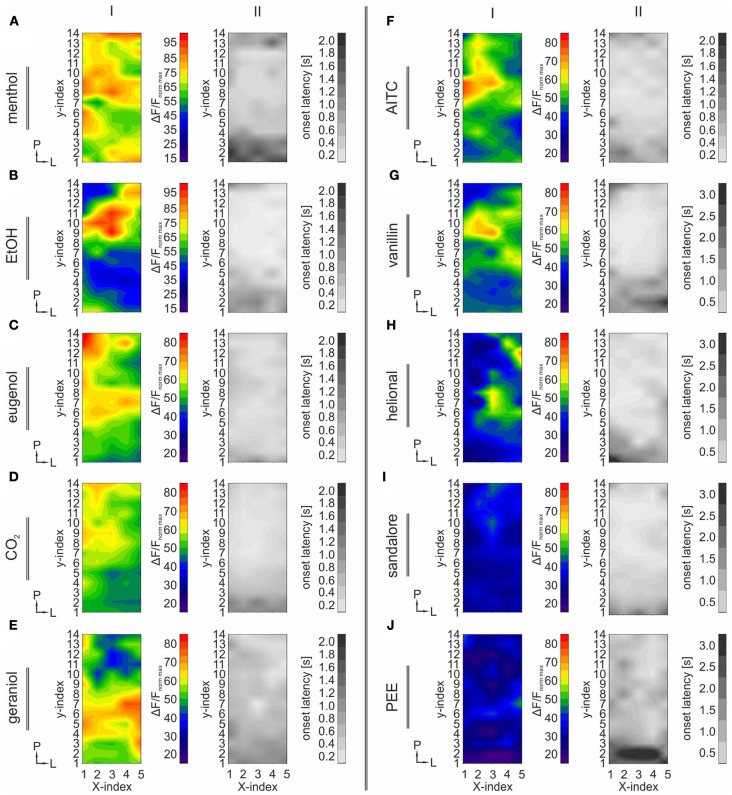
**TG regions with the strongest activity display the shortest onset latencies.** The figure is arranged in columns indicated by roman numerals (I–II). Each row refers to one nasally administered stimulus: menthol **(A)**, EtOH **(B)**, eugenol **(C)**, CO_2_ **(D)**, geraniol **(E)**, AITC **(F)**, vanillin **(G)**, helional **(H)**, sandalore **(I)**, and PEE **(J)**. I: Contour plots depicting the median activity (Δ*F*/*F*) obtained from 70 ROIs (Figure 2A) homogenously distributed across the TG, averaged across all animals, respectively (*n* = 4–29). X- and Y-indices refer to the X- and Y-indices in Figure 2A. Prior to median-calculation, values were normalized to the strongest response induced by CO_2_ administration within the same animal, respectively. Activity patterns display stimulus-specific differences regarding the spatial distribution and signal-intensity. Some substances, e.g., menthol elicited a diffuse pattern of activity, while other patterns e.g., caused by EtOH displayed one spot within postero-medial regions displaying the strongest activity. Signal intensity was strongly dependent on the stimulus used and the ganglionic area. II: Contour-plots depicting median onset latencies obtained from the 70 ROIs (Figure 2A). X- and Y-indices refer to the X- and Y-indices in Figure 2A. Onset latencies were highly stimulus dependent. In general, shortest onset latencies were observed in areas displaying the strongest signal intensities.

In order to generalize data from multiple animals, we measured maximum signal intensities (Δ*F*/*F*) and the respective onset latencies in 70 ROIs within activity maps obtained from single animals. The uniform distribution of these ROIs is depicted in Figure 2A (red marks). Each ROI covered an area of 300 μm^2^. To exclude staining-specific intensity differences across animals, activity within individual ROIs was normalized to the ROI value representing the strongest response to CO_2_, in the same animal (max-normalized). Finally, the datasets from multiple animals challenged with the same substance (containing signal intensities or onset latencies within these 70 ROIs), were averaged and median values of signal intensities and onset latencies were depicted as contour plots in Figures 3A–J (I and II).

**Figure 4:**
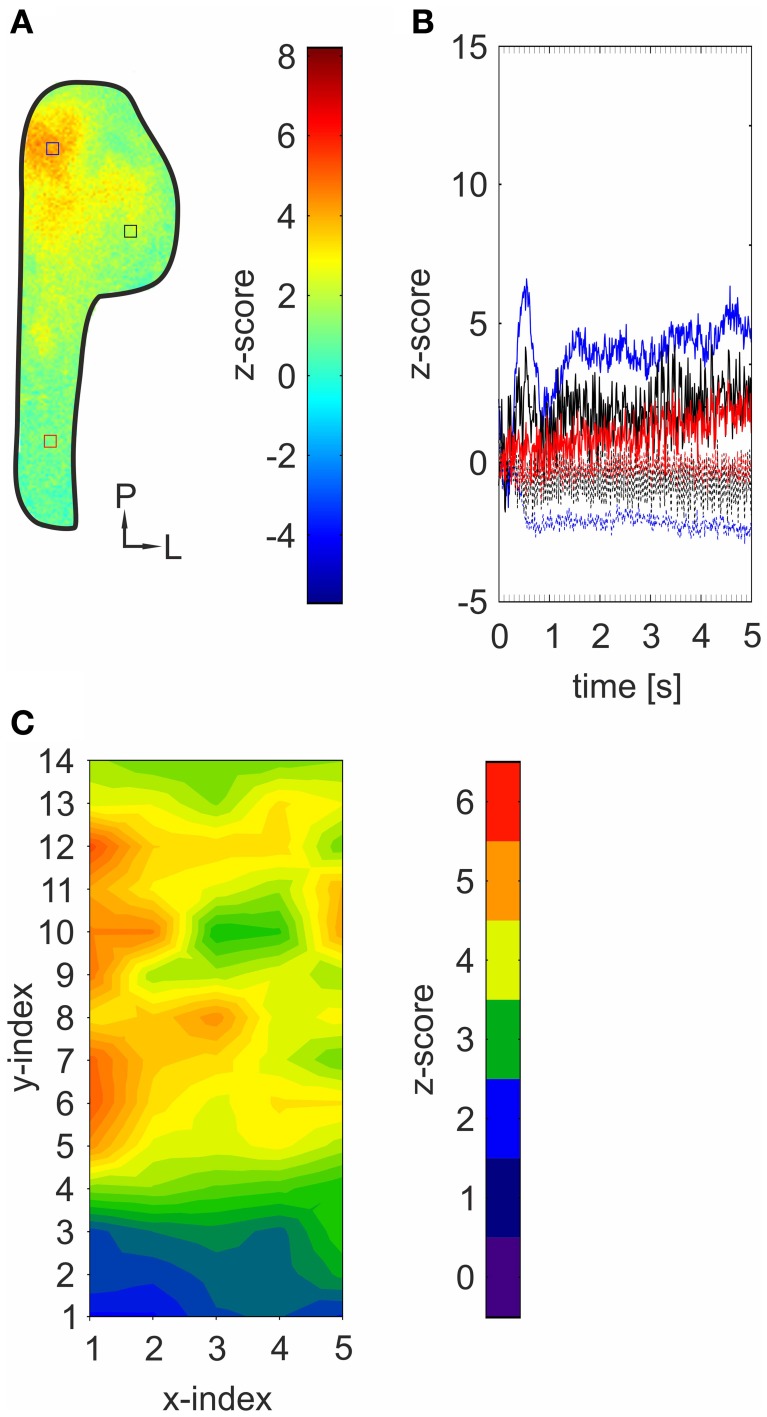
**Bioelectrical activity triggered by mechanical stimulation spreads across the TG. (A)** Representative *z*-score map (2000–4800 ms average) depicting an activity pattern induced by mechanical stimulation of the animal's nose derived from a single experiment. Colored squares indicate ROIs referring to local time courses depicted in **(B)**. **(B)** Local time course of *z*-score values obtained from the highlighted areas in the *z*-score map depicted in **(A)**. Strongest activity was monitored within postero-medial areas (blue). Lesser but still significant activity was detected in more anterior (red) and lateral (black) areas. Dotted lines: responses in the absence of any mechanical stimulation (blank-condition). **(C)** contour-plot depicting within-normalized activity measured within 70 ROIs across animals (*n* = 3). X- and Y-indices refer to the X- and Y-indices in Figure 2A. Within normalization: all values were normalized to the mean of the ten strongest ROI-values, respectively.

Generally, strongest activity was detected in postero-medial regions (Figures 1A–J, 3A–J; I). The exact localizations of these spots were stimulus dependent, resulting in different stimulus-specific activity patterns, which are further described in the following section. Concurrently, strongest signals (40.78–109.57%) monitored within these areas displayed the shortest onset latencies (90–380 ms), which highly varied between the activity evoked by individual substances (Figures 3A–J; II). These regions were recently reported to house mainly nasal TG neurons (Lazarov, 2002; Rothermel et al., 2011). Originating from these regions, activity seemed to expand to more anterior and lateral parts of the ganglion, still reaching significant values although with significantly lesser intensities (34.89–76.9%; *p* = 5.74 ^*^ 10^−5^ to 2.25 ^*^ 10^−3^) and longer latencies (500–3200 ms; *p* = 3.32 ^*^ 10^−6^ to 6.56 ^*^ 10^−3^). This activity spread was not restricted to nasal stimulation with volatile substances but also occurred upon mechanical stimulation of the animal's nose (*n* = 3) thereby excluding that it is based on retronasal stimulation (Figures 4A–C). Alike chemical stimulation, mechanically-evoked activity occurred within postero-medial regions of the TG, thereby further emphasizing that chemical stimulation activates sensory neurons innervating nasal regions (Figures 4A–C).

Together, we can show that all tested substances induced significant bioelectrical activity in the TG *in vivo* and could thereby confirm that VSDI can be used to monitor bioelectrical activity arising from the rat TG *in vivo*, evoked by volatile chemicals including vanillin (Rothermel et al., 2011). Differences between monitored activity patterns, signal intensities, and onset latencies of the detected bioelectrical activity will be analyzed separately in the following.

### Analysis of odorant-related stimulus-evoked activation patterns

Activity patterns elicited by nasal administration of the tested substances were monitored across a total of 29 animals. In order to ensure the stability of stimulus-specific patterns recorded across different animals, we used the CO_2_-evoked patterns from all animals and divided them into two groups. Then, all values measured within each single ROI of the animals from group 1 were compared to the corresponding values measured within animals of group 2 by performing Wilcoxon-ranksum-tests. At a level of *p* < 0.05, we observed only in 1/70 areas (at the antero-lateral edge of the TG) a significant discrepancy between the two groups, indicating the stability of the monitored signals across different animals in all other areas.

**Figure 5:**
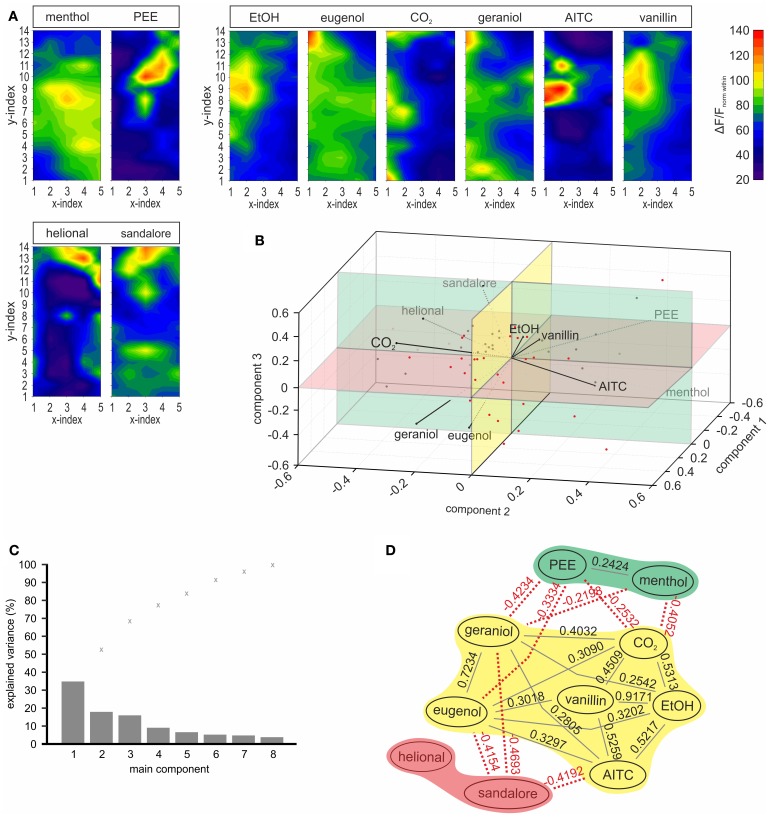
**Odorants can be sorted into three groups, by means of the evoked spatial response pattern. (A)** Contour-plots depicting within-normalized activity monitored upon stimulation with the ten substances tested in order to display the distribution of spatial activity independent from signal intensities. By means of calculation of spearman's rho and a PCA (see **B,C)** patterns were sorted in three different groups which are indicated by boxes above the contour-plots. The first group contained menthol and PEE, the second one EtOH, CO_2_, AITC, vanillin, eugenol, and geraniol, and the third one helional and sandalore. Within normalization: all values were normalized to the mean of the ten strongest ROI values, respectively. **(B)** PCA based on the within normalized activity patterns which contributed to the stimulus-sorting depicted in **(D)**. The three-dimensional analysis reveals correlation between menthol and PEE (group 1), between EtOH, vanillin, AITC, CO_2_, and to a lesser degree to eugenol and geraniol (group 2), as well as between helional and sandalore (group 3). **(C)** Bar diagram depicting the explained variance (%) by the single principle components, as well as the cumulative variance (x). >70% of variance is explained within the first 3 components, depicted in **(C)**. **(D)** Correlations between activity patterns evoked by the substances tested (within-normalized datasets were used for calculations). Connections between patterns indicate significant (*p* < 0.05) correlations. Black lines: positive correlations, red dotted lines: negative correlations. Spearman's rho indicated with each connection, indicates the dimension of the correlation. The three groups of substances were defined by calculation of Spearman's rho and the PCA **(B,C)** are highlighted in different colors (blue, yellow, green).

In order to characterize spatio/temporal dynamics of the response patterns, we aimed to analyze the spatial distribution of stimulus-evoked activity independent from absolute signal intensities. For this, all ROI values were normalized relative to the mean of the ten highest ROI values within the same condition (within-normalized). Afterwards, within-normalized datasets elicited by the same stimulus in different animals could be averaged and depicted as contour plots (Figure 5A).

We then tested, to which extend these within-normalized activity patterns correlate to each other. Therefore, we used two mathematical approaches, namely calculation of the Spearman's rank correlation coefficient [Spearman's rho (ρ)] and a PCA. For both calculations, we used the within-normalized 70 point datasets, respectively. These approaches allowed sorting the monitored activity patters into three different groups (Figure 5D). Group 1 (highlighted in green) contained menthol and PEE, group 2 (highlighted in yellow) was the biggest group and contained the substances EtOH, eugenol, CO_2_, geraniol, AITC, and vanillin, while group 3 (highlighted in blue) contained the substances sandalore and helional. Significant correlations (*p* < 0.05) between substances are represented in Figure 5D, which are indicated by lines between substances (gray: positive; red dotted: negative). The dimension of each correlation is given by the respective rank correlation coefficient (ρ). Correlations between stimulus-specific activity patterns were further substantiated by the performed PCA (Figures 5B,C). Again, the within-normalized and averaged stimulus-specific activity patterns were used for the calculation.

**Figure 6:**
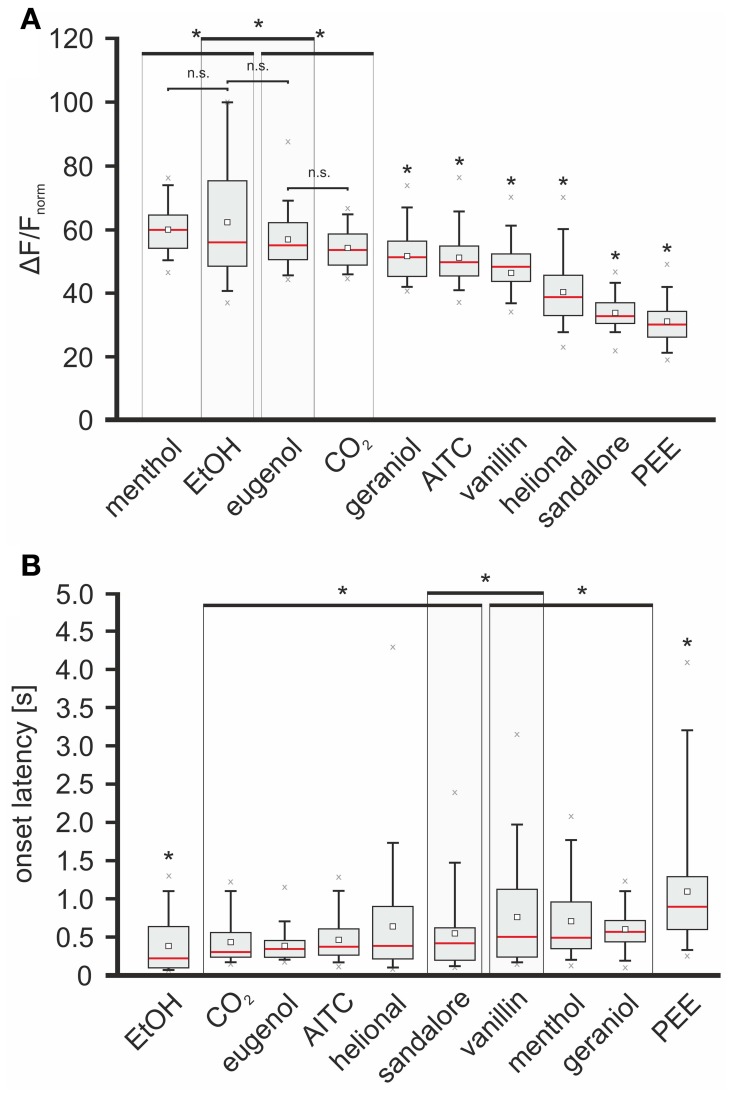
**Odorants trigger signals with specific intensities and onset latencies.** Red line, median; white square, mean; upper/lower edges of the gray boxes: 1st and 3rd quartile; whiskers, 5th and 95th percentile; x, outliers. Large boxes enclosing box plots indicate that max-amplitudes/onset latencies of enclosed box plots are not significantly different, although they differ from max-amplitudes/onset latencies of signals evoked by substances whose box plots lie outside these boxes (indicated by asterisks). **(A)** Box plot diagram depicting the median-intensity of signals monitored in response to nasal administration of the given substances within the 70 ROIs. Activity (Δ*F*/*F*) was normalized relative to the highest response evoked by nasal administration of CO_2_, respectively. With respect to the intensity of the evoked signals, substances were sorted in the following order (descending): menthol (59.97 +64.32/−54.38%), EtOH (55.89 +74.99/−48.37%), eugenol (54.83 +61.74/−50.71%), CO_2_ (53.68 +58.23/−48.67), geraniol (51.41 +56.09/−45.35%), AITC (49.46 +54.5/−45.29), vanillin (45.92 +51.94/−43.54%), helional (38.65 +45.03/−32.71%), sandalore (32.52 +36.43/−30.31 %), and PEE (29.86 +33.72/−26.05%). **(B)** Box plot diagram depicting onset latencies of signals induced by the administered substances. With respect to the onset latencies of the evoked signals, substances were sorted in the following order (ascending): EtOH (220 +615/−200 ms), CO_2_ (300 +550/−242.5 ms), eugenol (340 +445/−246.25 ms), AITC (370 +600/−272.5 ms), helional (390 +867.5/−225 ms), sandalore (420 +607.5/−212.5 ms), vanillin (505 +1096.25/−246.25 ms), menthol (515 +347.5/−947.5 ms), geraniol (570 +707.5/−442.5 ms), PEE (895 +1265/−600 ms).

Second, intensities of stimulus-specific signals were compared among each other by calculating significance levels between all datasets (Wilcoxon-ranksum-test; *p* < 0.05), containing all averaged max-normalized median values measured within the 70 ROIs, respectively. In this way, the tested substances could be sorted in the following order: menthol > EtOH > eugenol > CO_2_ > geraniol > AITC > vanillin > helional > sandalore > PEE (values can be derived from Figure 6A). Signals evoked by geraniol, AITC, vanillin, helional, sandalore, and PEE were significantly weaker than signals evoked by CO_2_, eugenol, EtOH, and menthol. No significant differences were found between the intensities of signals evoked by menthol and EtOH and between signals evoked by EtOH and eugenol although those evoked by menthol were significantly stronger than those evoked by eugenol. Furthermore, signals induced by menthol, EtOH, and eugenol were stronger than those evoked by CO_2_ (see Figure 6A). Next, we wanted to investigate how signal intensities correlate with onset latencies. Sorting the stimuli according to the means of the median onset-latencies revealed the following order: EtOH < CO_2_ < eugenol < AITC < helional < sandalore < vanillin < menthol < geraniol < PEE (values can be derived from Figure 6B). Significances were compared as previously described for the comparison of signal intensities. Onset latencies of signals evoked by EtOH were significantly shorter than those by the other stimuli. No significant differences were observed among latency times of signals evoked by CO_2_, eugenol, AITC, helional, and sandalore as well as between those of signals evoked by sandalore and vanillin. Furthermore, onset latencies of signals evoked by vanillin were not significantly different from the ones evoked by menthol and geraniol. Longest onset latencies were monitored upon application of PEE, which were significantly longer than those of signals evoked by all other substances tested (Figure 6B).

These results confirm the appearance of stimulus-specific patterns of bioelectrical activity at the level of the TG. Activity-patterns and signal-intensities seem to occur independent from each other [e.g., eugenol and geraniol elicit significantly correlating activity patterns (*p* = 1.51 ^*^ 10^−12^; ρ = 0.7234 (Figure 4B)] although with different intensities (*p* = 1.2 ^*^ 10^−4^), possibly indicating that the same group of sensory afferents is activated by the two substances but with different efficacies. Interestingly, we found no direct correlation between the intensity and the onset latency of stimulus-induced signals. Assuming that the onset latency of bioelectrical activity in the TG correlates with the perception of a sensation induced by a given substance, stimulus-specific onset latencies carry further information which is independent from stimulus-evoked activity patterns and the intensity of neuronal activity.

### Dose response dependency of signals arising from the TG

**Figure 7:**
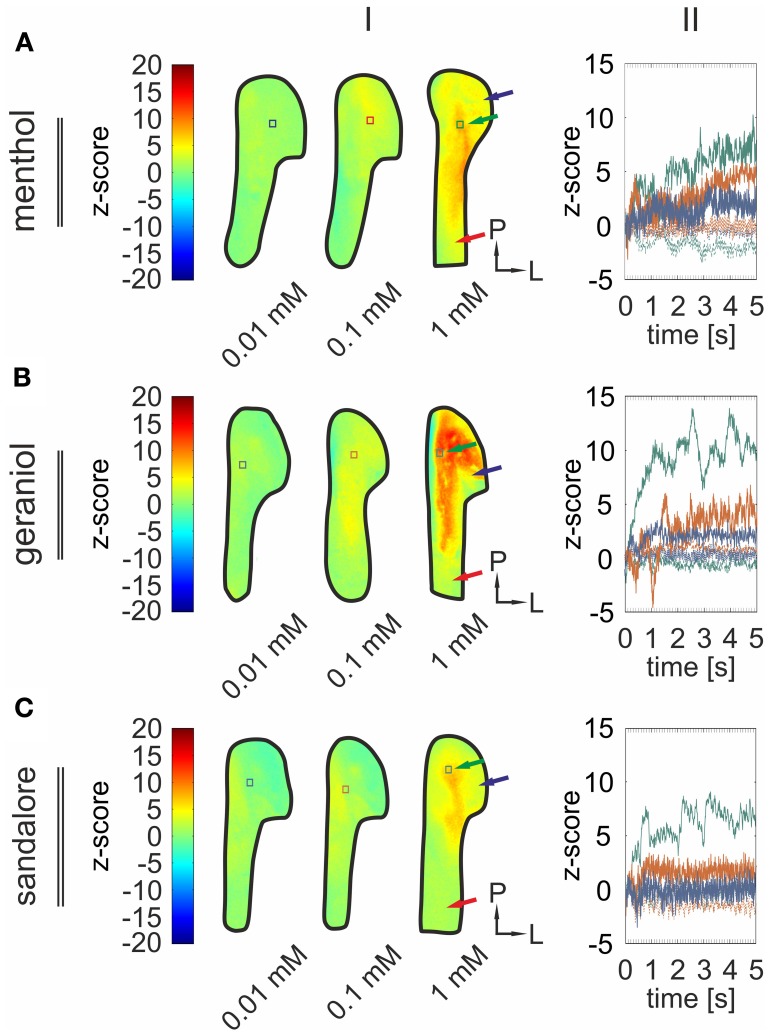
**Signal intensity diversifies with varying stimulus concentrations.** (I) Representative *z*-score maps (2000–4800 ms average) depicting stimulus-induced activity patterns in the TG derived from single animals upon nasal administration of menthol **(A)**, geraniol **(B)**, and sandalore **(C)** at varying concentrations (0.01, 0.1, and 1 mM). Colored squares indicate ROIs referring to local time courses depicted in II. (II) Local time course of *z*-score values obtained from the ROIs indicated in (I). Signal amplitudes increased along with increasing stimulus concentrations. Blue, signals monitored upon administration of the given substance at a concentration of 0.01 mM; red, of 0.1 mM; green, of 1 mM.

The previous findings revealed activity patterns characteristic for the monitored signals. Next, we wanted to investigate how the concentration of an administered stimulus correlates with the intensity of signals arising from the TG. Therefore, the olfactometer was equipped with aqueous solutions with various concentrations (0.01, 0.1, 1 mM) of menthol (*n* = 3–6), geraniol (*n* = 4–7), and sandalore (*n* = 4–5), respectively (Figure 7).

**Figure 8:**
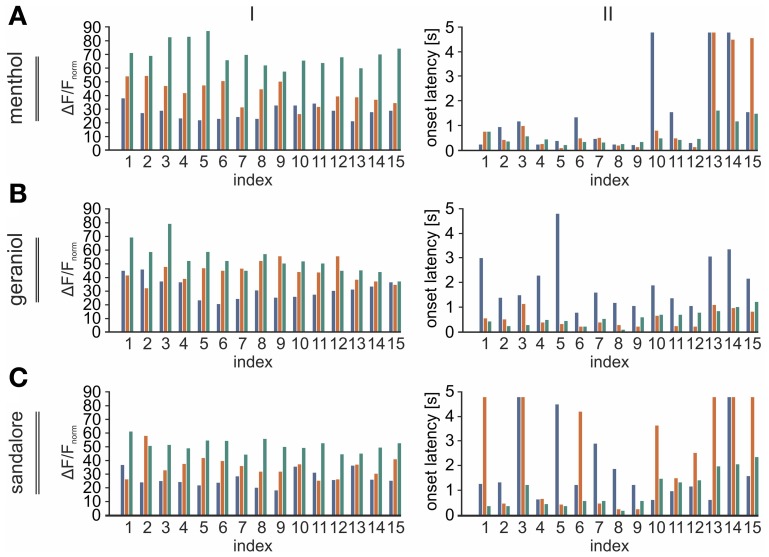
**Intensities and onset latencies of signals evoked by different stimulus-concentrations diversify among distinct regions of the TG. (A–C)** I: Bar diagrams depicting the activity (Δ*F*/*F*) monitored within the 15 ROIs (numbered 1–15; referring to Figure 2B) in response to different concentrations of the same substance (blue, 0.01 mM; red, 0.1 mM; black, 1 mM). Amplitudes were normalized relative to the strongest response to CO_2_ in the same animal. They display a strong concentration-dependency which was most prominent in postero-medial areas of the TG. II: Bar diagrams depicting onset-latencies of signals evoked by different concentration of the given substances in the 15 ROIs (numbered 1–15; referring to Figure 2B) averaged across animals (blue, 0.01 mM; red, 0.1 mM; green, 1 mM).

As described above, signal intensities (Δ*F*/*F*) were measured within 15 out of 70 ROIs uniformly distributed across the TG. These ROIs are indicated by numbers from 1 to 15 in Figure 2B. Data were normalized relative to the strongest ROI-value evoked by CO_2_ in the same animal. Again, datasets were averaged across animals and the respective median values were depicted as bar diagrams (Figure 8), thereby allowing the direct comparison of signal amplitude and onset latency within the same ROIs.

During these recordings, all substances evoked significant signals in a concentration dependent manner (Figure 7). Again, strongest activity was observed in postero-medial parts of the TG (black arrows) while signals monitored in more antero-medial (red arrows) and lateral regions (blue arrows) displayed smaller amplitudes (Figures 7, 8).

**Figure 9:**
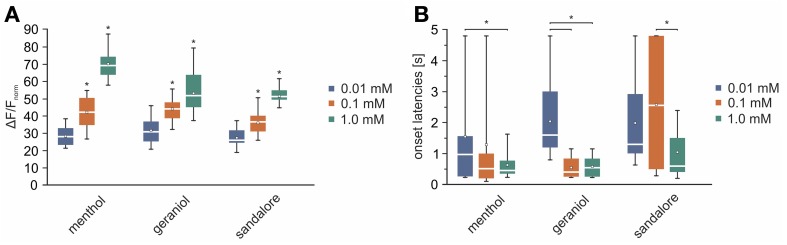
**Dose effect dependency of monitored signals. (A,B)** White line, median; white square, mean; upper/lower edges of the colored boxes (blue, 0.01 mM; red, 0.1 mM; green, 1 mM), 25th and 75th percentile; whiskers 5th and 95th percentiles; x, outliers. **(A)** Box plot diagram depicting the averaged and normalized activity induced by nasal administration of menthol (0.01 mM: 28.01 +23.15/−30.84%; 0.1 mM: 41.9 +48.71/−35.64; 1 mM: 59.97 +64.32/−54.38 %), geraniol (0.01 mM: 30.6 +36.4/−25.46%; 0.1 mM: 43.96 +47.19/−38.73%; 1 mM: 51.57 +57.78/−45.03%), and sandalore (0.01 mM: 25.71 +30.16/−24.38%; 0.1 mM: 36.37 +38.81/−31.48%; 1 mM: 50.96 +53.78/−49.34%) in all 15 ROIs (Figure 2B) across animals (*n* = 3–7). Signals increased significantly along with increasing concentrations of the stimuli (indicated by asterisks). **(B)** Box plot diagram depicting the averaged onset latencies of signals evoked by different concentrations of menthol (0.01 mM: 970 +1560/−285 ms; 0.1 mM: 510 +910/−235 ms; 1 mM: 455 +677.5/−352.5 ms), geraniol (0.01 mM: 1600 +2650/−1290 ms; 0.1 mM: 405 +757.5/−405 ms; 1 mM: 405 +757.5/−405 ms), and sandalore (0.01 mM: 1300 +2410/−1095 ms; 0.1 mM: 2560 +1470/−500 ms; 1 mM: 600 +1470/−445 ms). Onset latencies of signals decreased significantly with increasing stimulus concentrations.

On average, signals monitored upon application of 0.01 mM menthol significantly increased upon elevating the concentration to 0.1 mM menthol (*p* = 1.2 ^*^ 10^−6^) and to 1 mM (*p* = 3.39 ^*^ 10^−6^). The same applied to signals monitored upon application of geraniol (*p*_0.01/0.1_= 2.6 ^*^ 10^−4^; *p*_0.1/1_ = 0.014) and sandalore (*p*_0.01/0.1_ = 0.0011; *p*_0.1/1_ = 4.8 ^*^ 10^−3^) (values can be derived from Figure 9A). The increase of the amplitudes was accompanied by a decrease of the signal's onset latencies. Those of menthol-induced signals decreased significantly upon increasing the concentration from 0.01 to 1 mM (*p*_0.01/1_ = 0.0462). A similar decrease of onset latencies was observed upon increasing the concentration of geraniol from 0.01 to 0.1 mM (*p*_0.01/0.1_ = 1.2 ^*^ 10^−4^), although no further increase was evoked upon administration of 1 mM geraniol (*p*_0.1/1_= 0.5896). Onset latencies of signals evoked by 0.01 mM signals were not significantly different from those evoked by 0.1 mM sandalore (0.1; *p*_0.01/0.1_= 0.3757), although they significantly decreased upon administration of 1 mM sandalore (1 mM; *p*_0.1/1_ =0.0028) (values can be derived from Figure 9B).

These findings demonstrate that signal amplitude and onset latency highly depend on the concentration of nasally administered stimuli. Together with the previous findings, our results indicate that different characteristics of a given substance, namely kind and concentration, are already represented at the level of the TG. We show for the first time that this becomes apparent by stimulus-specific spatial activity patterns, differences in signal intensities and onset latencies, together forming a complex representation of volatile chemicals at the level of the TG.

## Discussion

By using a recently established protocol which allows monitoring of neuronal activity within the rat TG (Rothermel et al., 2011), we obtained new insights into the representation of volatile chemicals within this structure. We confirmed the occurrence of stimulus-specific activity patterns in the TG which were suggested previously (Rothermel et al., 2011). While the previous study focused mainly on the establishment of the surgery and verified the accordance between VSD-signals and neuronal activity within the TG, we have now used this technology to more thoroughly characterize activity patterns in the TG triggered by nasal administration of a large number of compounds in different concentrations. We first monitored the patterns of spatial activity caused by the administration of a set of ten different volatile chemicals. Upon investigating the monitored patterns irrespective of signal intensity and onset latencies, we could discriminate three different types of pattern. Based on their data set, Rothermel et al. (2011) suggested that the strength of a given stimulus was related to a specific activity pattern (e.g. EtOH and citral were rated as “strong” and “weak” trigeminal stimuli. Our analysis of several concentrations of the substances tested, revealed that while the activity patterns are characteristic for the compounds applied, signal intensity depends on the concentrations of administered stimuli. We conclude that activity patterns and signal intensities carry independent information characterizing both, the particular stimulus and its concentration. Further information may be derived from the onset latencies of the signals. A similar latency rank based encoding of odorants was verified at the level of mitral/tufted cells in the vertebrate olfactory bulb, which seems to contain all information needed by higher brain regions to identify odors and their concentrations (Junek et al., 2010).

The comparison of signal intensities and onset latencies is problematic, since the concentrations of EtOH, CO_2_, and AITC differed from the other substances, all of which were used at concentrations of 1 mM. However, some striking observations became apparent in the consideration of only some of those substances, namely menthol, eugenol, geraniol, helional, vanillin, sandalore, and PEE. Several compounds with strong signal intensities also displayed short onset latencies (e.g., CO_2_ and eugenol). On the other hand, menthol elicited significantly stronger signals than for instance AITC, while the onset latencies of AITC-evoked signals were substantially shorter than those evoked by menthol. Based on the foregoing we conclude that a particular stimulus is represented in the TG by (1) a specific pattern, (2) the intensity, and (3) the onset latency of the pattern. These three aspects may jointly provide information about the nature and the concentration of the specific compound.

Incomplete knowledge exists on the mechanisms that may be involved in the detection of volatile substances by trigeminal nerve terminals and processing the information to form the patterns that we have recorded. Fundamentally distinct mechanisms are likely to exist for the detection of compounds. EtOH affects a long list of voltage- and ligand-gated channels including TRPV1 (Trevisani et al., 2002). Other substances like menthol or AITC tend to affect single receptors, often members of the TRP-channel family (McKemy et al., 2002; Peier et al., 2002; Bandell et al., 2004; Jordt et al., 2004), of which TRPA1 seems to also mediate the detection of CO_2_ (Wang et al., 2010). Other substances like geraniol or eugenol activate multiple channels from this family (Yang et al., 2003; Behrendt et al., 2004; Stotz et al., 2008). Among others, TRP channels on trigeminal sensory fibers seem to be crucially involved in the detection of chemical volatiles outside the OS. This raises the question whether different expression patterns of these channels within the TG are underlying the observed activity patterns. It is well-accepted that subpopulations of trigeminal neurons express different sets of receptor proteins (Belmonte and Viana, 2008). Additionally, it was shown that different populations of TG neurons that innervate distinct facial areas reveal different sensitivities for capsaicin and menthol (Damann et al., 2006). However, only one study characterized the detailed distribution of neurons expressing TRPA1, TRPV1, and TRPM8 across the whole rat TG by *in situ* hybridization (Kobayashi et al., 2005). These authors reported that TRPM8 is mainly expressed in neurons of the maxillary branch, while TRPV1 and TRPA1 are homogenously expressed across the entire TG. Interestingly, while menthol activates TRPM8 (McKemy et al., 2002; Peier et al., 2002), the observed menthol-evoked activity patterns were not restricted to neurons of the maxillary branch but strong activity was observed across large ganglionic areas. In contrast, nasal administration of the TRPA1 agonist AITC, caused strong bioelectrical activity which was confined to postero-medial regions of the ganglion. This region contains mainly somata of nasal fibers which was shown by viral tracing (Rothermel et al., 2011). Topographically, somata of the mandibular branch are housed within the posterolateral portion of the TG, while cell bodies of the ophthalmic branch are localized anteromedially, and those of the maxillary branches are interposed in-between (López de Armentia et al., 2000; Lazarov, 2002). Interestingly, the observed activity patterns were not restricted to these regions. This indicates that formation of the observed activity patterns is not exclusively based upon a certain distribution of TG neurons within the ganglia but appears to be more complex involving further mechanisms.

We conclude that, while TRP-channels certainly contribute to the shape of stimulus-specific activity patterns, our data suggest an involvement of additional factors. This might include thus far unidentified receptors but also paracrine or direct cell to cell communication in the trigeminal ganglia.

Further modulation of chemically evoked activity patterns in the TG may result from other events activating the TS that occur simultaneously with the chemically evoked activation, e.g., mechanical stimulation, occurring during the motion of breathing. Several studies demonstrated a strong relation between intranasal mechanical stimulation with other sensory inputs, for instance the OS (Deschênes et al., 2012). Just sniffing was shown to trigger activity in the human olfactory cortex (Sobel et al., 1998) and to affect the ability to localize different odors (Frasnelli et al., 2009). Sniffing results in different activity patterns in the olfactory bulb in rodents, and seems to modulate the encoding of different odors (Verhagen et al., 2007). This possibly provides an adaptive filter suppressing background odors. The modulation of olfactory information by sniffing may result from increased attention. It may, however, also be the consequence of a direct mechanical effect on olfactory sensory neurons or of mechanical stimulation of trigeminal fibers (Grosmaitre et al., 2007). These findings indicate a complex modulation of nasal chemosensory input by simultaneous mechanical stimulation. Although it is not known whether sniffing and/or mechanical activation impacts trigeminal chemosensation, it seems likely that sniffing or other mechanical stimuli affect chemically evoked trigeminal sensations. In this study, we aimed to isolate chemically evoked trigeminal activity, irrespective of mechanical or thermal stimulation and attempted to minimize interference of mechanical stimuli, for what we used an olfactometer and reverse tracheotomy. The interference of the isolated trigeminal odorant signals measured here with other sensory stimuli remains open for further investigations.

All substances tested in our experiments evoked bioelectrical activity within the TG upon nasal administration. While vanillin and PEE had previously been viewed as exclusively olfactory stimuli (Doty et al., 1978; Radli and Wysocki, 1998; Cometto-Muñiz et al., 2005), it was shown later that vanillin also activates TRPV3 in concentrations >10 mM (Xu et al., 2006). Unpublished data from our group showed that vanillin directly activates recombinant rTRPV1 and rTRPA1 and recent evidence indicated that vanillin activates the TS *in vivo* (Rothermel et al., 2011). Therefore, it may not come as a surprise that vanillin causes trigeminal activity *in vivo*, but the mechanisms underlying the activity evoked by sandalore and PEE are unknown. Both compounds triggered no direct effect on the membrane potential of cultured TG neurons (unpublished data).

Several studies verified a close interplay between the OS and the TS and one possible interaction site is the olfactory epithelium (Bouvet et al., 1987; Jacquot et al., 2004). Stimulation of the OS may increase trigeminal sensations (Livermore et al., 1992; Roscher et al., 1997). One potential mediator of this effect is ATP, released by olfactory sensory neurons upon activation (Spehr et al., 2004). Extracellular ATP may then activate purinoreceptors, expressed in a subpopulation of trigeminal sensory fibers (Lazarov, 2002). The involvement of ATP explains our finding that raising the sandalore concentration from 0.01 to 0.1 mM led to increased signal intensities, but longer onset latencies. Spehr et al. (2004) reported that co-administration of different odorants with ATP reduced the maximum and rise time of P2X_2_-mediated currents in cultured trigeminal neurons. At higher concentrations, sandalore may trigger an ATP release from olfactory neurons, such that higher concentrations still revoke stronger trigeminal activity, but onset latencies are prolonged due to the influence of extracellular ATP. Together, an interaction with the OS might thereby contribute to the overall perception of chemical volatiles by the TS. Since anosmia is not necessarily connected to damage of the olfactory epithelium but might also be caused by disruption of the olfactory nerve or failures in higher brain regions, this may also be the case in subgroups of anosmic patients. Additionally, several other cells of neuronal as well as non-neuronal origin (e.g., keratinocytes) were shown to release ATP upon stimulation, wherefore they are further possible sources (Bodin and Burnstock, 2001; Mandadi et al., 2009).

Generally, the strongest signals with the shortest onset latencies were monitored in postero-medial parts of the TG, containing mainly somata of nasal fibers (Lazarov, 2002; Rothermel et al., 2011). Spatial distribution of mechanically evoked activity revealed the strongest overlap with the pattern triggered by chemicals causing mainly burning and stinging sensations such as EtOH, CO_2_, AITC, or eugenol. It is tempting to speculate that these substances activate nociceptive neurons that also contribute to the development of painful sensations such as the response to mechanical stimulation (Viana, 2011). Originating from postero-medial regions, activity was spreading to more anterior and lateral areas of the TG, in which we observed smaller but still significant activity with prolonged onset latencies. A similar dispersion was observed after mechanical stimulation, excluding retronasal stimulation of oral sensory afferents as a source for this activity. Several studies suggested a cross excitation between different cells within sensory ganglia (Amir and Devor, 1996, 2000; Ulrich-Lai et al., 2001; Oh and Weinreich, 2002), possibly caused by intraganglionic release of different transmitter substances [for instance ATP, calcitonin gene-related peptide (CGRP), or substance *P*]. Both peptides are well-known to be released upon activation of different subpopulations of TG neurons, some of them positive for TRPA1 and TRPV1 (Matsuka et al., 2001; Ulrich-Lai et al., 2001; Thalakoti et al., 2007; Kunkler et al., 2011; Edelmayer et al., 2012). However, it has not yet been possible to verify the existence of cross excitation within sensory ganglia *in vivo.* Further experiments will have to clarify whether the observed spreading of activity is indeed based on cell–cell communications within the ganglion and to understand its underlying mechanisms and functions. Our results indicate a complex representation and possibly an early processing of volatile chemicals within the TG. The representation of specific substances through activity patterns in the TG, as well as their signal intensities and onset latencies, is likely to involve several mechanisms, rather than being a simple relay station transmitting peripheral signals to the brain. Although we are far from understanding the detailed representation of volatile chemicals in the TG, our results represent relevant contribution to unraveling the TS, which is instrumental to medical indications such as migraine or trigeminal neuralgia.

### Conflict of interest statement

The authors declare that the research was conducted in the absence of any commercial or financial relationships that could be construed as a potential conflict of interest.
